# Daturine

**Published:** 1865-02

**Authors:** 


*Daturine.*—M. Jobert de Lamballe has for some time past been employing, as a mydriatic, in his opthalmic practice, and as a substitute for the various preparations of belladonna, a simple solution of daturine. The facts upon which he founds his preference for this drug are the following: Daturine is three times as strong as atropine and its salts, and may consequently be used in proportionately smaller doses. When introduced between the eyelids no pain is produced, neither is the vision dimmed, as when belladonna is employed. The effects produced are, moreover, more reliable and constant, as well as more durable.

